# Secondary School Staff Engagement With a School-Based Physical Activity and Sedentary Behavior Initiative Website: Implementation Trial During National Scale-Up

**DOI:** 10.2196/95250

**Published:** 2026-10-29

**Authors:** Anna Fitriani, Ana María Contardo Ayala, Harriet Koorts, Samuel K Lai, Jo Salmon

**Affiliations:** 1 Institute for Physical Activity and Nutrition, School of Exercise and Nutrition Sciences, Deakin University, 221 Burwood Highway, Burwood, Melbourne, Victoria, 3125, Australia, 61 392446333

**Keywords:** teacher training, physical activity, sedentary behavior, web-based intervention, real world, scale-up

## Abstract

**Background:**

Online platforms are increasingly used to deliver training and implementation resources for large-scale, school-based physical activity initiatives, and understanding behavioral engagement with these platforms is important because it provides an early indicator of initiative uptake; yet, few studies have examined engagement during real-world scale-up.

**Objective:**

This study examined school staff engagement with the *TransformUs* website and its associated factors during the first 22 months of national scale-up in secondary schools.

**Methods:**

As part of an implementation trial, engagement metrics were obtained from the *TransformUs* website (October 2023 through August 2025) using Google Analytics, and user and school characteristics were derived from registration data on the *TransformUs* website and the *My School* Australia database. Engagement metrics were summarized descriptively (eg, number and frequency of pages accessed, time spent on pages, and content-specific metrics), and associations between staff- and school-level characteristics and engagement outcomes were examined using multivariable linear and logistic regression models.

**Results:**

A total of 200 secondary school staff from 6 Australian states and 2 territories registered for the *TransformUs* website, of whom 110 were tracked by Google Analytics. Users included school leaders, physical education (PE) teachers, and non-PE teaching staff, with most users being female and working in government schools. Overall engagement was predominantly characterized by single-visit use, with a median of 1 (IQR 1-2) session, and approximately one-third of users accessed only the registration page. Across the study period, users viewed a median of 5 (IQR 2-9) pages and spent a median of 8.4 (IQR 5.2‐13.1) minutes on the website. Page reach was highest for general information and teaching resources pages. Compared with schools in Victoria, engagement duration was longer in the Northern Territory (β=2.98 min; *P*=.01) and South Australia (β=4.05 min; *P*=.01), while schools with high student-teacher ratios had shorter engagement than those with low student-teacher ratios (β=–3.11 min; *P*=.01). Compared with low-achieving schools, schools with medium academic achievement had greater odds of accessing implementation resources (odds ratio 4.74, 95% CI 1.26‐17.85; *P*=.02).

**Conclusions:**

Engagement with the *TransformUs* website during secondary school national scale-up reflected the opportunistic and self-directed nature of engagement with online initiatives in real-world settings. Dissemination during scale-up should not end when users receive the materials but should also ensure meaningful engagement with web-based resources. Future research should measure more meaningful dimensions of engagement and examine school- and user-level factors to better understand determinants of engagement and inform scale-up strategies.

## Introduction

Over the last decade, the proportion of Australian adolescents meeting physical activity guidelines has remained low with little improvement [1,2], while excessive screen-based sedentary behavior has continued to increase [3]. These behaviors are associated with adverse physical [4], mental [5], and cognitive outcomes [6]. Multicomponent, whole-of-school physical activity interventions are recommended to address these risks [7,8], and scaling effective initiatives is necessary to maximize population impact [9]. Given that adolescence coincides with the secondary school years, secondary schools provide a critical setting for large-scale implementation.

Online training and resources are increasingly used in state and national physical activity initiatives because they can reach geographically dispersed schools while reducing the costs and burden of face-to-face delivery [10-14]. These resources are commonly disseminated through websites and promoted via channels such as social media, teacher magazines, webinars, conferences, and email [15]. Websites function as a platform for delivering training and implementation resources to support program adoption and implementation [16,17]. Their flexible and self-directed format allows school staff to access materials at a time and pace that suits their needs [18], consistent with recommendations to provide autonomy in training-based health initiatives [19]. Website-based training has also been shown to be acceptable, appropriate, and feasible among secondary school staff [16].

Engagement with online training encompasses affective, cognitive, and behavioral dimensions [20]. Assessing engagement is important because it influences knowledge and skill acquisition, which are key antecedents of successful implementation and scale-up [21-23]. Although implementation is not directly observable through website analytics, tools such as Google Analytics provide objective indicators of behavioral engagement, including website visits, time spent on pages, and resources accessed [24,25]. These data can help identify which resources attract attention and where engagement declines, informing future scale-up efforts.

Little is known about how school staff engage with website-based training and resources when school-based physical activity initiatives are scaled up in real-world settings. While studies conducted under research trial conditions have reported promising engagement outcomes [16,17], evidence from other fields suggests that website engagement is often lower in real-world contexts [26]. User characteristics and contextual factors are recognized determinants of engagement with online professional learning [27]. Factors such as user role, experience, prior exposure to an initiative [16,17,27], workload and capacity constraints [28], the time needed to learn and prepare for a new initiative [29], and support and prioritization at both school and regional levels [28] may all influence engagement.

Since its launch in October 2023, *TransformUs Secondary* has been disseminated nationally through coordinated efforts between the research team and 16 partner organizations [15] directing school staff to the *TransformUs* website [30]. Understanding engagement with these resources is important because engagement represents a precursor to knowledge and skill acquisition and an indicator of initiative uptake [23,31]. Accordingly, this study aimed to (1) describe school staff engagement with the *TransformUs* website secondary school components across Australia and (2) examine associations between the user- and school-level factors with website engagement.

## Methods

### Intervention and Study Context

*TransformUs Secondary* is an adaptation of the primary-school *TransformUs* initiative [32,33], tailored for adolescents in secondary school settings [34]. The primary-school version of *TransformUs* has demonstrated efficacy in Victoria, reducing children’s sedentary time, increasing physical activity, and supporting healthy weight outcomes [32]. *TransformUs Secondary* consists of 6 components (ie, active academic lessons, active breaks, active homework, health fact sheets, active environments, and peer support) and is being evaluated in a type II hybrid effectiveness-implementation trial [34]. To support scale-up, training modules and initiative resources for school staff were made available online through the *TransformUs* website.

Dissemination involved collaboration with 16 partner organizations across Australian states and territories (eg, departments of education and health, professional teaching associations, and peak bodies), which were provided with a toolkit containing adaptable promotional materials (eg, flyers and social media posts). A full description of these dissemination strategies has been published elsewhere [15]. During the first 16 months post-launch, dissemination occurred through both research team-led and partner-led activities [15], with partners using a range of strategies tailored to their local context (Fitriani et al [15]). The dissemination and implementation trial of *TransformUs Secondary* was prospectively registered with the Australian New Zealand Clinical Trials Registry (ACTRN12622000600741). While the registered trial did not originally include a website engagement analysis, this component was included as an add-on within the broader *TransformUs Secondary* implementation evaluation. The website engagement analysis was used to assess the reach and adoption of disseminated web-based resources. The reporting of this implementation study followed the Guidelines and Checklist for the Reporting on Digital Health Implementations (i-CHECK-DH; Checklist 1) [35].

### TransformUs Website: Structure and Content

The *TransformUs* website [30] served as the primary platform for training and resource delivery. Following registration, teachers received unique login credentials and could access the website at any time during the study period (October 10, 2023, through August 21, 2025). The platform did not require a structured navigation sequence, allowing users to freely explore content according to their needs. The website comprised 21 unique pages, which were grouped into 5 categories based on content type: (1) General Information, (2) Registration, (3) Training, (4) Teaching Resources, and (5) Active Environment. General Information included introductory and informational content designed to promote awareness of the initiative and encourage registration. Registration referred to the enrollment page used to create a user account. Training included support materials, instructional guides, and planning resources to assist with implementation. Teaching Resources provided practical classroom materials and examples of activities, while Active Environment focused on supporting schools in developing active school environments and related policies.

### Participant and Recruitment

*TransformUs Secondary* was disseminated to all Australian secondary schools (n=1453) and their associated staff (n=230,592) across all states and territories [34]. Schools included government schools (operated by state or territory authorities), Catholic schools, and independent schools (including those affiliated with specific religious denominations, educational philosophies, or operating as stand-alone entities) [36]. Dissemination materials distributed by partner organizations contained a hyperlink directing school staff to the *TransformUs* website. School staff who were interested in the initiative self-registered online at no cost. Registration required completion of an online form that collected school and staff characteristics, which were used in this study.

### Data Collection and Measures: Website Engagement Metrics

Website engagement data were extracted from Google Analytics 4 [37] and filtered to include only sessions by users who had registered for *TransformUs Secondary* within the defined study period (October 10, 2023, through August 21, 2025). Because the *TransformUs* website also hosts materials for other components of the initiative (eg, *TransformUs Primary*, *TransformUs All Abilities*, and *TransformUs Higher Education*) and participants in the *TransformUs Secondary* effectiveness trial, filters were applied to isolate activity on pages relevant only to the secondary school implementation trial and excluded nonregistered or unrelated traffic. All filtering procedures were implemented in collaboration with Deakin University’s Digital Infrastructure and Cloud Services team to ensure accurate extraction.

For Aim 1, key engagement metrics were collected, comprising *overall engagement level* (ie, user-level measures of website use, including total sessions, total page views, total duration, and average page views and average session duration) and *page-level engagement patterns* (ie, content-specific measures of engagement across website pages, capturing time spent on pages, and page reach); these metrics were also summarized by time registration to examine differences across user cohorts, as detailed in Multimedia Appendix 1. Overall engagement level provides a summary of general user activity that can be benchmarked across studies, whereas page-level engagement patterns capture content-specific behaviors and the breadth of engagement across website pages, including which pages were accessed, how frequently, and for how long.

For Aim 2, we operationalized web engagement through specific metrics derived from overall and page-level usage data: (1) average time spent on training, accessing teaching resources, and accessing active environment pages (minutes) and (2) the likelihood (odds) of users visiting training, teaching resources, and active environment pages (“Yes”/“No”), as summarized in Multimedia Appendix 1. The first metric captures interaction with training and resources underpinning knowledge and skill acquisition, while the second reflects engagement with key implementation content. These metrics were defined as the primary indicators of engagement for the *TransformUs* platform and were used to examine which staff and school characteristics were associated with these outcomes. The selection and reporting of metrics for both Aim 1 and Aim 2 were guided by the *AMUsED* (A Framework for Analyzing and Measuring Usage and Engagement Data) framework, which provides a structured approach to capturing and interpreting user interaction with digitally based interventions [25] (Checklist 2).

### School and Staff Characteristics

School and staff characteristics as independent variables were obtained from registration data and publicly available sources, including the Australian *My School* website [38]. School characteristics collected from registration data include age, gender, years of experience in education, role, the presence of a school physical activity policy, and the channel through which they first heard about *TransformUs Secondary. Staff roles* were hierarchically classified: school leaders (principals, assistant principals, well-being coordinators, and other leadership roles), physical education (PE) teachers, and non-PE teaching staff (classroom teachers, preservice teachers, casual relief teachers, and specialist teachers). Staff holding multiple roles were assigned to the highest-ranking category, prioritizing leadership over PE teaching, and PE teaching over non-PE roles. The presence of a school physical activity policy was recorded as “Yes”/“No,” indicating whether the school had documented guidance on students’ physical activity.

School data obtained from the *My School* website included school geographic location, school sector, and school type. School geographic location was classified by state/territory (eg, Victoria and New South Wales) and remoteness (major cities; regional, combining inner and outer regional; and remote, combining remote and very remote) [39]. School sector was classified as government or nongovernment, including Catholic schools and independent schools [36]. School type was classified as secondary (delivering secondary education), combined (delivering both primary and secondary education), or special (catering to students with special needs) [36]. Socioeconomic status of the school’s location was measured using the Socio-Economic Indexes for Areas (SEIFA) score [40], derived from census data. School academic achievement was represented by the average National Assessment Initiative—Literacy and Numeracy (NAPLAN) scores [36]. Student-teacher ratio was calculated as the number of enrolled students divided by full-time equivalent teaching staff. For analysis, school socioeconomic status, academic achievement, and student-teacher ratio were categorized into low (≤25th percentile), medium (26-75th percentile), and high (>75th percentile) groups based on the distribution of their respective values across the study sample.

### Data Analysis

For univariate analyses (Aim 1), school and staff characteristics, overall engagement levels, and page-level engagement patterns were summarized using descriptive statistics. Categorical school and staff characteristics were presented as percentages, while numerical engagement metrics were summarized using medians and IQRs to account for their nonnormal distributions. Given that registration dates varied across users due to different launch timing in each state, univariate analyses of engagement metrics (total sessions, total page views, and total duration) were stratified according to the number of months since each individual registration, with each user included in one of these categories: 0 to 6 months, 7 to 12 months, 13 to 18 months, or ≥19 months.

Associations between staff and school characteristics and engagement outcomes (Aim 2) were analyzed using linear regression models for continuous outcomes (average time spent on training, accessing teaching resources, and accessing active environment pages) and logistic regression models for binary outcomes (whether users visited training, teaching resources, and active environment pages [“Yes”/“No”]). For each outcome, separate multivariable regression models were fitted for each predictor, with all models adjusted for the same set of covariates (teacher age, teacher gender, school remoteness, school SEIFA status, and time since registration). The number of observations included in regression models varied across predictors due to missing data, ranging from 39 to 52 participants. Categorical independent variables, including ordinal covariates, were entered into the regression models using dummy coding. Multicollinearity was assessed using the variance inflation factor, with all values below 5, indicating no serious multicollinearity. Statistical significance was set at *P*<.05 for all analyses. For linear regression models, diagnostic checks were performed to assess model assumptions, including examination of residual distributions, residual vs fitted plots, and influential observations using Cook’s distance. Sensitivity analyses were conducted by repeating linear regression models after excluding observations identified as potentially influential, which did not alter the overall interpretation of the findings. All analyses were conducted using Stata/SE (version 18.0; StataCorp LLC).

### Ethical Considerations

This study was conducted with the approval of the Deakin University Human Research Ethics Committee (Project IDs: 2021‐269 and HEAG-H 32_2021). The study used deidentified registration and website engagement data collected from school staff who registered for the *TransformUs Secondary* initiative. As outlined in the approved registration form and privacy statement, participants were informed that the information collected at registration could be used for program evaluation and program-related research in addition to program administration. In accordance with the approved ethics protocol, the analysis of deidentified registration and website analytics data did not require individual informed consent.

All data were stored securely in encrypted files accessible only to approved researchers. No participant compensation was provided.

## Results

### Staff (Website Users) and School Characteristics

Between October 10, 2023, and August 21, 2025, 200 secondary school staff registered for the *TransformUs* website. Of these, 110 users could be tracked by Google Analytics, whereas the remaining 90 were not tracked, likely due to consent-related browsing issues such as declining cookies or using browsers that block website tracking. A comparison of user and school characteristics between included and excluded users showed no statistically significant differences between the 2 groups. Among included users, most were women, older than 40 years, had fewer than 21 years of experience in education, and worked in government schools. Users represented a range of staff roles and were distributed across all Australian states and territories. The most frequently reported channels through which staff learned about *TransformUs Secondary* were government departments or authorities, the education sector, and nongovernment organizations (Multimedia Appendix 2).

### Overall Engagement Level

Table 1 presents overall website engagement among users and by time since registration. Overall, users typically accessed the website once, with 5 total page views and 8.4 minutes of website use. Per session, users viewed a median of 4 (IQR 2-6) pages and spent a median of 6.2 (IQR 3.7-10.6) minutes on the website. Engagement patterns for total sessions and average page views per session were generally similar across registration cohorts, while some differences were observed, including higher total page views among users registered for 19 or more months and higher total and average session duration among those registered within the previous 6 months.

**Table 1.** Website engagement levels, overall and by time since registration^a^.

Engagement metric	All users, median (IQR)	0‐6 months, median (IQR)	7‐12 months, median (IQR)	13‐18 months, median (IQR)	≥19 months, median (IQR)
Sessions	1 (1-2)	1 (1-1)	1 (1-1)	1 (1-2)	1 (1‐2.5)
Page views	5 (2-9)	4 (2-6)	5 (2-8)	5 (2-9)	7 (4-10)
Duration (min)	8.4 (5.2‐13.1)	12.6 (7.5‐17.7)	9.5 (5.3‐14.3)	7.5 (4.3‐12.9)	9.6 (6.1‐15.5)
Pages per session	4 (2-6)	4 (2-6)	4 (2-5)	3 (2-6)	4 (2.8‐5.5)
Session duration (min)	6.2 (3.7‐10.6)	12.6 (7.5‐17.7)	5.8 (3.9‐9.5)	6.1 (3.4‐9.8)	6.5 (3.5‐11.1)

a Data are presented as medians (IQRs) due to nonnormal distribution of engagement metrics and are stratified by time since registration to account for variability in exposure duration across users.

### Page-Level Engagement Patterns

Table 2 presents page-level engagement patterns across page categories and user roles. Notably, one-third of all users reached only the registration page without viewing any additional page categories. Among pages that were accessed, variation in time spent on pages and in page reach was observed across page categories. General Information pages had relatively short engagement duration but the highest overall reach, whereas Teaching Resources and Training showed longer engagement durations but lower reach. Active Environment had the lowest overall reach and relatively short engagement duration. At subpage level, Active Break had the longest engagement duration among subpages and the highest overall reach, followed by How It Works and Active Lesson. Engagement breadth varied by staff role, with PE teachers showing the greatest tendency to explore multiple additional page categories beyond registration.

**Table 2.** Time spent on page and page reach, overall and by school role^a^.

Page and sub-page visited	Time spent on page (min), median (IQR)	Page reach, n (%)			
		School leaders (n=35)	PE^b^ teachers (n=34)	Non-PE teaching staff (n=41)	All (N=110)
General information	1.0 (0.1‐47.3)	19 (54.3)	20 (58.8)	18 (43.9)	57 (51.8)
How it works	0.7 (0.1‐14.1)	14 (40)	11 (32.4)	17 (41.5)	42 (38.2)
Meet the team	0.7 (0.5‐1.4)	0 (0)	2 (5.9)	3 (7.3)	5 (4.5)
For teachers	1.0 (1.0‐1.0)	1 (2.9)	0 (0)	0 (0)	1 (0.9)
For parents	5.3 (0.6‐9.9)	0 (0)	0 (0)	2 (4.9)	2 (1.8)
Active learning	0.7 (0.3‐12.6)	6 (17.1)	10 (29.4)	5 (12.2)	21 (19.1)
Informing research	1.5 (0.4‐47.3)	3 (8.6)	6 (17.6)	4 (9.8)	13 (11.8)
Training	1.35 (0.08‐110.95)	6 (17.1)	7 (20.6)	6 (14.6)	19 (17.3)
How-to: active homework	0.55 (0.50‐0.60)	0 (0)	1 (2.9)	1 (2.4)	2 (1.8)
How-to: active lesson	0.74 (0.03‐35.40)	4 (11.4)	3 (8.8)	3 (7.3)	10 (9.1)
How-to: active break	1.15 (0.08‐69.50)	3 (8.6)	7 (20.6)	5 (12.2)	15 (13.6)
How-to: active environment	2.02 (0.20‐3.83)	0 (0)	1 (2.9)	1 (2.4)	2 (1.8)
How-to: health lesson	0.51 (0.30‐0.72)	4 (11.4)	3 (8.8)	3 (7.3)	10 (9.1)
Planning (state matter 1, 2, 3)	1.73 (0.80‐2.67)	0 (0)	1 (2.9)	1 (2.4)	2 (1.8)
Support (eg, getting started, inclusion)	1.03 (0.50‐2.39)	1 (2.9)	1 (2.9)	1 (2.4)	3 (2.7)
Teaching resources	1.98 (0.08‐60.9)	15 (42.9)	18 (52.9)	23 (56.1)	56 (50.9)
Active lesson	0.83 (0.02‐51.27)	7 (20)	11 (32.4)	10 (24.4)	28 (25.5)
Active break	2.37 (0.08‐43.75)	12 (34.3)	14 (41.2)	17 (41.5)	43 (39.1)
Active homework	0.88 (0.30‐1.47)	0 (0)	0 (0)	2 (4.9)	2 (1.8)
Health lesson	0.68 (0.17‐2.00)	7 (20.0)	11 (32.4)	10 (24.4)	28 (25.5)
Active environment	0.97 (0.52‐6.35)	0 (0)	4 (11.8)	2 (4.9)	6 (5.5)
Combination of page categories					
Registration only	N/A^c^	11 (31.4)	10 (29.4)	12 (29.3)	36 (32.7)
Registration +1 additional page category	N/A	12 (34.3)	8 (23.5)	14 (34.1)	26 (23.6)
Registration +2 additional page categories	N/A	8 (22.9)	9 (26.5)	12 (29.3)	32 (29.1)
Registration +3‐4 additional page categories	N/A	4 (11.4)	7 (20.6)	3 (7.3)	15 (13.6)

a Page-level engagement is presented as median (IQR) time spent per page and percentage page reach, stratified by school role and time since registration to account for differences in exposure duration across users.

b PE: physical education.

c N/A: not applicable.

### Associations Between User- and School-Level Factors With Website Engagement

Multivariate analyses are presented in Tables 3 and 4, with sample sizes ranging from 39 to 52 across models. Table 3 shows associations between staff and school characteristics and the average time spent engaging with the Training, Teaching Resources, and Active Environment pages. Compared with users from schools with a low student-teacher ratio, those in schools with a high ratio spent significantly less time on these pages (β=–3.11 min; *P*=.01). Compared with users from schools in Victoria, those in the Northern Territory (β=2.98 min; *P*=.01) and South Australia (β=4.05 min; *P*=.01) spent significantly more time engaging with training and resources.

**Table 3.** Separate multivariable linear regression models of the associations between teacher and school characteristics and the average session time spent on the training, teaching resources, and active environment pages (β, 95% CI).

Staff and school characteristics^a^	β (95% CI)	*P* value
Role (reference: teaching staff)		
School leader	–1.72 (–4.02 to 0.58)	.14
PE^b^ teacher	–1.90 (–3.99 to 0.17)	.07
Years working in education (reference: <21)		
21‐40	–2.35 (–4.78 to 0.08)	.06
School sector (reference: government)		
Catholic	–1.69 (–4.12 to 0.73)	.17
Independent	–1.10 (–4.44 to 2.05)	.46
School type (reference: standard)		
Combined/special	–0.36 (–2.68 to 1.95)	.75
Availability of school PA^c^ policy (reference: yes)		
No	1.75 (–1.67 to 5.16)	.31
Do not know	1.96 (–0.31 to 4.24)	.09
School academic achievement (average NAPLAN^d^ score), (reference: low)		
Medium	1.62 (–1.37 to 5.26)	.24
High	1.96 (–5.30 to 3.40)	.66
Student/teacher ratio (reference: low)		
Medium	1.96 (0.31 to 4.24)	.09
High	–3.11 (–5.51 to –0.70)	.01
School location (reference: Victoria)		
New South Wales	3.97 (–2.05 to 9.99)	.19
Northern Territory	2.98 (0.65 to 5.31)	.01
Queensland	–0.78 (–3.53 to 1.96)	.57
South Australia	4.05 (1.88 to 6.22)	.01
Source of information (channel), (reference: government departments)		
School communities	0.95 (–2.83 to 4.72)	.61
Nongovernment associations	–0.29 (–3.24 to 2.65)	.84
Others (eg, media and university)	2.77 (–1.76 to 7.31)	.22

a Separate multivariable linear regression models were fitted for each predictor. All models were adjusted for the same set of covariates: teacher age, teacher gender, school remoteness, school Socio-Economic Indexes for Areas (SEIFA) status, and time since registration.

b PE: physical education.

c PA: physical activity.

d NAPLAN: National Assessment Initiative—Literacy and Numeracy.

**Table 4.** Separate multivariable logistic regression models of the associations between teacher and school characteristics and the odds of accessing any training, teaching resources, or active environment pages (OR^a^, 95% CI).

Staff and school characteristics^b^	OR (95% CI)	*P* value
Role (reference: teaching staff)		
School leader	0.65 (0.23-1.86)	.42
PE^c^ teacher	0.78 (0.27-2.30)	.65
Years working in education (reference: <21)		
21‐40	2.23 (0.74-6.74)	.16
School sector (reference: government)		
Catholic	2.55 (0.72-9.04)	.15
Independent	0.80 (0.19-3.28)	.76
School type (reference: standard)		
Combined	0.88 (0.32-2.40)	.81
Availability of school PA^d^ policy (reference: yes)		
No	0.36 (0.09-1.50)	.16
Do not know	0.63 (0.22-1.80)	.38
School academic achievement (average NAPLAN^e^ score), (reference: low)		
Medium	4.74 (1.26-17.85)	.02
High	3.52 (0.60-20.77)	.16
Student/teacher ratio (reference: low)		
Medium	0.85 (0.27-2.70)	.78
High	0.61 (0.18-2.06)	.42
School location (reference: Victoria)		
New South Wales	0.18 (0.01-4.26)	.29
Northern Territory	0.85 (0.20-3.62)	.83
Queensland	0.51 (0.12-2.21)	.37
South Australia	0.67 (0.22-2.05)	.48
Source of information (channel) (reference: government departments)		
School communities	1.57 (0.38-6.46)	.53
Nongovernment associations	3.18 (0.93-10.93)	.07
Others (eg, media, university, and Google search)	3.83 (0.80-18.36)	.09

a OR: odds ratio.

b Separate multivariable logistic regression models were fitted for each predictor. All models were adjusted for the same set of covariates: teacher age, teacher gender, school remoteness, school Socio-Economic Indexes for Areas (SEIFA) status, and time since registration.

c PE: physical education.

d PA: physical activity.

e NAPLAN: National Assessment Initiative—Literacy and Numeracy.

Table 4 shows the associations between staff and school characteristics and the odds of accessing any of the Training, Teaching Resources, or Active Environment pages. Compared with users from schools with low academic achievement, those from medium-achieving schools had more than 4 times odds of visiting Training, Teaching Resources, and Active Environment pages (odds ratio 4.74, 95% CI 1.26‐17.85; *P*=.02).

## Discussion

### Principal Findings

This study examined secondary school staff’s engagement with the *TransformUs* website, a whole-of-school physical activity and sedentary behavior initiative, and the factors associated with engagement. Overall engagement level showed that engagement was concentrated in a single visit rather than through repeated use, as indicated by a median of 1 (IQR 1-2) session per user, one-third of users accessing only the registration page, and consistent patterns across registration cohorts. Page-level engagement pattern demonstrated that engagement was concentrated on informational and teaching resource pages, with variation in engagement breadth across staff roles. In addition, state- and school-level factors (ie, student-teacher ratio, location/state, and academic achievement) were associated with engagement with the implementation resources (Training, Teaching Resources, and Active Environment pages).

The brief engagement with the *TransformUs Secondary* website suggests opportunistic interactions that may represent a typical mode of engagement with digital interventions in real-world settings [41]. These patterns differed from those observed in the Physical Activity for Everyone (PA4E1) school–based physical activity initiative implemented under controlled trial conditions, which demonstrated substantially higher repeated engagement across sessions, page views, and duration [16]. Unlike PA4E1, which was embedded within a structured intervention with guided support and in-school champions [16], *TransformUs Secondary* functioned as a voluntarily accessed, nationally disseminated resource platform, reflecting self-directed use in real-world settings [34,41]. However, it should be noted that engagement pattern in this study reflects only behavioral metrics and does not capture other dimensions of engagement such as cognitive or emotional engagement [20], meaning it should not be interpreted as nonmeaningful engagement given that not all dimensions were captured.

Although users often accessed the website only once, within-session engagement (eg, page views per session and session duration) was comparable to that observed in the PA4E1 study, indicating potential opportunities to support repeat engagement. Web-based resources such as online professional learning are designed to provide flexible, self-paced access to implementation assistance over time [18] to accommodate teachers’ time constraints [42]. As such, initial school-based digital interventions may be characterized by limited repeat visitation, potentially reflecting flexibility in use and staff time constraints, where some return visits may have occurred outside the study period. This highlights the need for ongoing monitoring and strategies to encourage teachers to revisit websites, particularly training and implementation resources [43-45].

While overall engagement metrics provide a broad overview of website use, page-level analysis helps identify which content was accessed and by whom. Engagement was concentrated on General Information and Teaching Resources pages, suggesting that users primarily accessed brief introductory content and practical classroom materials. In contrast, Training and Active Environment pages received limited attention, potentially because these activities are perceived as complex, time-intensive, and requiring organizational change [46,47]. Although self-guided online interventions allow users to access content according to their needs, they may also encourage selective use and limited exposure to key intervention components [24]. Therefore, providing initial guidance or requiring completion of key activities may help enhance engagement [24].

A more important consideration is whether engagement with key implementation resources was sufficient to support implementation in practice. Although this could not be assessed directly, the relatively short duration of engagement warrants attention, as time spent on a website is often used as an indicator of use [25]. Teaching Resources and Training pages contain relatively dense implementation content, including instructional videos, yet the average engagement duration was brief. While time spent on a page does not necessarily reflect content consumption or application, these findings raise questions about whether users achieved meaningful cognitive engagement beyond surface-level scanning [48]. This may reflect known limitations of self-directed online training, including passive participation, early dropout, and reduced opportunities for skill development [49]. Brief engagement may also limit users’ ability to process complex online content [50]. To address these challenges during scale-up, previous research has recommended interactive features, opportunities for collaboration and instructor support, prompts, guided activities, and tools that help users navigate and synthesize content [18,49,50].

Across page categories and subpage reach, PE teachers and non-PE teaching staff showed broadly similar engagement patterns, whereas school leaders demonstrated narrower engagement. This likely reflects differing roles in program dissemination [22]. School leaders may access websites primarily to review resources for oversight and coordination, whereas teachers engage more extensively with implementation materials for classroom delivery. These findings suggest that website content may be used differently across staff roles and highlight the importance of tailoring website design to users’ responsibilities and preferences.

Consistent with evidence that usage of online training and resources during scale-up is highly context-dependent [51], this study found that engagement varied by both school- and state-level characteristics. However, the absence of direct measures of key individual-level factors, such as workload, time constraints, motivation, digital literacy, and leadership support, which are known to influence engagement with online resources [27,42,52], limits the interpretation of these associations. As such, observed relationships should be considered indirect reflections of underlying contextual influences.

Shorter engagement durations in schools with higher student-teacher ratios may reflect multiple unmeasured mechanisms in this study, such as individual factors (eg, perceived workload imbalance and limited capacity leading to burnout) [53] and school-level factors (eg, competing priorities that reduce time available to engage with the *TransformUs* website). Our previous work similarly found that teachers face competing priorities when deciding whether to engage with *TransformUs* resources [15]. State-level differences in engagement, including longer engagement with training and implementation resources in South Australia and the Northern Territory, may reflect variation in dissemination approaches, partner support, and physical activity policy contexts [54]. Similarly, greater engagement in medium-achieving compared with low-achieving schools may reflect broader contextual differences associated with educational disadvantage and regional or remote settings, where teachers often face competing demands, limited support, and professional isolation [55,56]. Taken together, these findings highlight the need for future research to integrate both website-level and individual- and organizational-level determinants of engagement to better explain variation in website use and inform targeted scale-up strategies.

### Strengths and Limitations

A key strength of this study is its real-world scale-up context, allowing website engagement to be captured under routine, uncontrolled conditions. This provides a novel contribution to dissemination and implementation research, as engagement with digital resources at scale is rarely reported in school-based physical activity interventions [57]. By examining both overall and page-level engagement, the study demonstrates that simple aggregate web metrics are insufficient to characterize user interaction. Operationalizing website engagement as a scale-up outcome also advances implementation science by positioning digital resource use as an indicator of adoption, with implications for optimizing platform design and delivery.

This study has several limitations. Users whose website use could not be tracked due to technical reasons, such as not providing consent for website tracking, were excluded from the analyses. However, comparisons of user and school characteristics showed no statistically significant differences between included and excluded users. Engagement was assessed using behavioral metrics (eg, duration and page views), which may not capture deeper forms of engagement, such as learning or implementation. In addition, relevant user- and school-level factors (eg, leadership support, user needs, preferences, and perceptions) were not measured, limiting the explanation of observed patterns. Furthermore, separate regression models were used to explore potential associations between individual staff and school characteristics and engagement outcomes. Although this approach was appropriate given the exploratory nature of the analyses and the available sample size, it does not account for the increased risk of type I error arising from multiple statistical tests and the potential interrelationships between predictors. Therefore, these findings should be considered hypothesis-generating rather than confirmatory. Finally, unequal follow-up periods due to variation in registration timing may have affected the assessment of longer-term engagement.

### Conclusions

Overall engagement with the *TransformUs* website during national scale-up was characterized by predominantly single visits, concentrated use of general information and teaching resource pages, and associations with some school- and state-level factors. These findings suggest that web-based dissemination strategies during scale-up should extend beyond ensuring that resources reach intended users and should instead support meaningful and sustained engagement with these resources. Additionally, understanding how engagement varies across users and contexts is critical for maximizing the impact of web-based scale-up efforts.

## Supplementary material

10.2196/95250 Multimedia Appendix 1 Website engagement metrics.

10.2196/95250 Multimedia Appendix 2 Comparison of school staff characteristics between included and nonincluded users.

10.2196/95250 Checklist 1 iCHECK-DH checklist.

10.2196/95250 Checklist 2 AMUsED checklist.

## Abbreviations

AMUsED: A Framework for Analyzing and Measuring Usage and Engagement Data
i-CHECK-DH: Guidelines and Checklist for the Reporting on Digital Health Implementations
NAPLAN: National Assessment Initiative—Literacy and Numeracy
PA4E1: Physical Activity for Everyone
PE: physical education
SEIFA: Socio-Economic Indexes for Areas
