# Supplementary material for: Secondary School Staff Engagement With a School-Based Physical Activity and Sedentary Behavior Initiative Website: Implementation Trial During National Scale-Up
Source: JMIR Form Res. 2026 Oct 29;10:e95250. doi: 10.2196/95250 (PMC13718533; doi:10.2196/95250)
Supplement: Supplementary file 1 [file formative-v10-e95250-s001.docx]

## **Multimedia Appendix 1.** Website engagement metrics

| Metric | Definition | Unit |
| --- | --- | --- |
| **Overall Engagement Level (Aim 1)** | |  |
|  |  |  |
| Total sessions | The total number of continuous periods of user interaction with the website (e.g. clicking, opening pages, and watching videos), ended after 30 minutes of inactivity. | Count |
|  |  |  |
| Total pageviews | The total number of times a webpage was loaded across all sessions, including repeat views of the same page within or across sessions. For example, if a user navigated to a page, visited another page, and then returned to the original page, each visit was counted as a separate pageview. | Count |
|  |  |  |
| Total duration | The cumulative time (minutes) each user spent on the website across all sessions during the study period. | Minutes |
|  |  |  |
| Average pageviews per session | The mean number of pages viewed per session, calculated by dividing the total number of pageviews by the total number of sessions. Repeated views of the same page within a session were included. | Count |
|  |  |  |
| Average session duration | The mean length of time users spent on the website per session, calculated across all sessions during the study period. | Minutes |
|  |  |  |
| **Page-level Engagement Pattern (Aim 1)*** | |  |
|  |  |  |
| Time spent on page | Time is calculated based on the interval between page interactions and is aggregated at the user level. | Minutes |
|  |  |  |
| Page reach | The proportion of users who accessed a specific page, sub-page, or combination of pages at least once during the study period. Page reach also informed a derived measure of engagement breadth, categorised by the number of page categories accessed per user (registration only, +1, +2, or +3–4 categories). Page reach was calculated for each staff group (school leaders, PE teachers, and non-PE teaching staff) and overall, across all users. | % |
|  |  |  |
| **Engagement Outcomes (Aim 2)** | |  |
|  |  |  |
| Average time spent on training, teaching resource, and active environment pages | Mean duration a user spends actively viewing training, teaching resources, or active environment pages. | Minutes |
|  |  |  |
| The likelihood of users visiting training, teaching resource, and active environment pages | The probability that a registered user accessed at least one page containing training modules, teaching resources, or the active environment page during the study period. | Yes/No |

Note: This table summarizes engagement metrics used in the study; additional explanation of key measures is provided in the main text to support interpretation. *A page refers to a primary website category (General Information, Training, Teaching Resources), while a subpage denotes a specific content page nested within these categories (e.g., How-to modules under Training and Active Break under Teaching Resources).
