# Supplementary material for: Secondary School Staff Engagement With a School-Based Physical Activity and Sedentary Behavior Initiative Website: Implementation Trial During National Scale-Up
Source: JMIR Form Res. 2026 Oct 29;10:e95250. doi: 10.2196/95250 (PMC13718533; doi:10.2196/95250)
Supplement: Supplementary file 2 [file formative-v10-e95250-s002.docx]

**Multimedia Appendix 2.** Comparison of School Staff Characteristics Between Included and Non-Included Users

| Characteristics | Included n/N (%) | Non-Included n/N (%) | P value³ |
| --- | --- | --- | --- |
| Gender |  |  |  |
| Female | 78/102 (76.5) | 68/86 (79) | 0.47 |
| Male | 24/102 (23.5) | 17/86 (20) |  |
| Prefer not to say | 0/102 (0.0) | 1/86 (1) |  |
| Age |  |  |  |
| 21–30 | 22/101 (21.8) | 18/84 (21) | 0.10 |
| 31–40 | 19/101 (18.8) | 29/84 (35) |  |
| 41–50 | 33/101 (32.7) | 16/84 (19) |  |
| 51–60 | 23/101 (22.8) | 17/84 (20) |  |
| 61+ | 4/101 (4.0) | 4/84 (5) |  |
| Years in education |  |  |  |
| 0–10 | 36/99 (36) | 43/83 (52) | 0.09 |
| 11–20 | 31/99 (31) | 19/83 (23) |  |
| 21–30 | 20/99 (20) | 17/83 (21) |  |
| 31–41 | 12/99 (12) | 4/83 (5) |  |
| Staff role |  |  |  |
| Leadership | 35/110 (31.8) | 33/90 (37) | 0.15 |
| PE teacher | 34/110 (30.9) | 17/90 (19) |  |
| Classroom teacher/staff | 41/110 (37.3) | 40/90 (44) |  |
| School sector |  |  |  |
| Catholic | 19/110 (17.3) | 7/90 (8) | 0.13 |
| Government | 78/110 (70.9) | 73/90 (81) |  |
| Independent | 13/110 (11.8) | 10/90 (11) |  |
| State |  |  |  |
| ACT | 0/110 (0.0) | 1/90 (1) | 0.42 |
| NSW | 2/110 (1.8) | 1/90 (1) |  |
| NT | 18/110 (16.4) | 19/90 (21) |  |
| QLD | 15/110 (13.6) | 16/90 (18) |  |
| SA | 22/110 (20.0) | 12/90 (13) |  |
| TAS | 0/110 (0.0) | 2/90 (2) |  |
| VIC | 52/110 (47.3) | 39/90 (43) |  |
| WA | 1/110 (0.9) | 0/90 (0) |  |
| PA policy availability |  |  |  |
| No | 11/94 (12) | 12/74 (16) | 0.63 |
| Yes | 60/94 (64) | 47/74 (64) |  |
| Don't know | 23/94 (25) | 15/74 (20) |  |
| Channel through which staff learned about the initiative |  |  |  |
| School communities | 14/83 (17) | 15/63 (23) | 0.07 |
| Non-government orgs | 24/83 (29) | 13/63 (20) |  |
| PA peak bodies | 18/83 (22) | 13/63 (20) |  |
| School leader association | 5/83 (6) | 0/63 (0) |  |
| Preventive health org | 1/83 (1) | 0/63 (0) |  |
| Government authorities | 33/83 (40) | 26/63 (39) |  |
| Education sector | 32/83 (39) | 23/63 (35) |  |
| Health sector | 1/83 (1) | 3/63 (5) |  |
| Others | 12/83 (15) | 9/63 (14) |  |
| School type |  |  |  |
| Secondary | 84/110 (76.4) | 69/90 (77) | 0.71 |
| Combined | 23/110 (20.9) | 20/90 (22) |  |
| Special | 3/110 (2.7) | 1/90 (1) |  |
| SEIFA¹ |  |  |  |
| ≤25^th^ percentile | 29/110 (26.4) | 22/90 (24) | 0.86 |
| >25 - ≤75^th^ percentile | 52/110 (47.3) | 46/90 (51) |  |
| >75^th^ percentile | 29/110 (26.4) | 22/90 (24) |  |
| NAPLAN² |  |  |  |
| ≤25^th^ percentile | 22/110 (20.0) | 21/90 (23) | 0.46 |
| >25 - ≤75^th^ percentile | 47/110 (42.7) | 43/90 (48) |  |
| >75^th^ percentile | 41/110 (37.3) | 26/90 (29) |  |
| Student/teacher ratio |  |  |  |
| ≤25^th^ percentile | 23/110 (20.9) | 24/90 (27) | 0.33 |
| >25 - ≤75^th^ percentile | 50/110 (45.5) | 44/90 (49) |  |
| >75^th^ percentile | 37/110 (33.6) | 22/90 (24) |  |

**Note:** ¹Socio-Economic Indexes for Areas. ²National Assessment Program – Literacy and Numeracy. Differences between groups were assessed using Pearson chi-square tests. ³Fisher's exact test was used where expected cell counts were small. No statistically significant between-group differences were observed.
