# Supplementary material for: Secondary School Staff Engagement With a School-Based Physical Activity and Sedentary Behavior Initiative Website: Implementation Trial During National Scale-Up
Source: JMIR Form Res. 2026 Oct 29;10:e95250. doi: 10.2196/95250 (PMC13718533; doi:10.2196/95250)
Supplement: Supplementary file 4 [file formative-v10-e95250-s004.docx]

## Checklist for the AMUsED Framework

| *Stage 1* | | | | | | | | | | |  |
| --- | --- | --- | --- | --- | --- | --- | --- | --- | --- | --- | --- |
| Familiarisation with the data – identifying variables | | | | | | | | | | |  |
| Generic questions by data type | | | Intervention: *TranformUs Secondary* | | | | Intervention: *Internet Dr* | | | |  |
| 1. Intervention characteristics. Data for intervention architecture and content. | | | | | | | | | | |  |
| 1.1 Workflow. Intervention structure and expected participant interaction and navigation through the intervention. | | | | | | | | | | |  |
| How many logins/sessions are available? | | | Not structured as formal sessions; participants access the platform as needed to:   1) Register: Create an account and complete a registration form with personal and school demographics. 2) Log in: Access professional learning modules and resources (optional, no sequence or time limit). | | | | *Not structured as sessions, access intended during an RTI.* | | | |  |
| When are they available? | | | *All content is available at any time and can be accessed freely once participants have an account to log in. No structured prompts; participants access the platform ad hoc and are encouraged to explore content via dissemination efforts.* | | | | *All content available at every access. Prompted logins at: baseline, 5 x 4-weekly interim questionnaires, & follow-up questionnaires. Then during illness and 48 hour follow-up.* | | | |  |
| Are new sessions released depending on time elapsed or task-completion? | | | *No; all content is available immediately and can be accessed freely once participants log in.* | | | | *No.* | | | |  |
| Are there limitations on the availability of the intervention? | | | No. | | | | *No.* | | | |  |
| Is the purpose of a session to collect self-report measures and/or use the intervention? | | | No. | | | | *Questions are asked after login to ascertain user’s purpose – either to use intervention for illness or to complete interim questionnaires.* | | | |  |
| When is the intervention considered to be finished? | | | *TransformUs Secondary* is an ongoing, real-world program with no fixed end. Participants can access the platform and resources as long as they wish, and intervention use depends on individual engagement rather than a set study period. | | | | *The study finishes after the follow-up questionnaire at 24 weeks. The intervention was finished when users no longer wished to access it.* | | | |  |
| What prompts are used to encourage usage (e.g. emails, texts, notifications) and when are they sent? | | | Dissemination efforts (e.g. social media, teacher webinars, newsletters) were primarily timed at the start of each school term, with additional mid- and end-term activities, to encourage teachers to register and engage with the website. | | | | *Email prompts for 4 weekly data collection.*  *Additional emails are sent after logging in during illness to prompt repeat login after 48 hours.* | | | |  |
| Does the intervention contain ‘tunneled’ (compulsory) sequences of pages which users have to view to move forward? | | | No. | | | | *‘Doctor’s Questions’ component is tunneled as symptoms are assessed before providing illness management advice.* | | | |  |
| Are users able to select linked components they wish to view, and avoid others? | | | Yes; users could freely select any components they wished to view and were not required to access other content. | | | | *There are 3 linked menu components available from the home page.* | | | |  |
| 1.2. Content. Content available within the pages of the intervention. | | | | | | | | | | |  |
| What are the components available? | | | | The website included two main components:   1) General information, such as ‘About Us,’ ‘What Is It,’ and ‘News & Research’; and 2) Resources, including professional learning, teaching materials, and active environment resources. | | | | *‘Doctor’s Questions’. Asks about symptoms & provides advice recommending either: self-management, phoning NHS Direct, or seeking immediate medical attention. ‘Treatment Options’. Advice on coping with symptoms: without medication, with medication, and boosting immune system. ‘Common Questions’. 2 sets of FAQs: ‘Ask the Internet Dr’ - medical questions about illness and treatment, ‘Common myths about Colds and Flu’ - general questions about illness beliefs.* | | | |
| What is the aim of each component and are they based on underlying theoretical constructs? | | | | 1) General information builds trust and credibility based on Diffusion of Innovations Theory 2) Resources foster motivation and engagement based on Self-Determination Theory | | | | *Doctor’s Questions and Common Questions support users who are unsure if their symptoms are serious and whether they need medical treatment. Based on Leventhal’s Common Sense Model of Self-regulation of health and illness* [3] *to increase understanding of illness. Treatment Options supports management of distressing symptoms. Based on Bandura’s Social Cognitive Theory* [4] *to increase self-efficacy.* | | | |
| In what order is it anticipated the components will be used? | | | | No specific order. | | | | *Doctor’s Questions first so as to check symptoms.* | | | |
| What interactive features are available (e.g. forums, videos, printable information)? How long should they take to complete? | | | | Videos: Short clips (~1.5 minutes each)  Frequently Asked Questions (FAQs)  Downloadable resources | | | | *Videos available: Welcome video on home page: 1 minute, 23 seconds, glands: 48 seconds, meningitis/septicaemia: 1 minute, 1 second, video sinusitis: 30 seconds. Printable material available but log-data doesn’t record whether it was used.* | | | |
| Are all components/features available to all users throughout the intervention or are some tailored for specific times or users? | | | | All components and features were accessible to all users at any time; no content was tailored to specific users or time points | | | | *Doctor’s Questions and Treatment Options are split into cough, sore throat, runny nose & fever.* | | | |
| Which pages are for collecting self-report measures or for administrative purposes | | | | None | | | | *Separate list available for content by individual page.* | | | |
| Are there specific pages to mark the start and end of sessions? | | | | *Home page is the first page visited.* | | | | *Home page is the first page visited.* | | | |
| Which pages contain BCTs (e.g. information, planning, feedback) and what are they? | | | | *See components listed previously* | | | | *See components listed previously* | | | |
| In which sessions are they available? | | | | Ad hoc access (available to all users), allowing users to freely access resources at any time | | | | *N/a* | | | |
| Can specific BCTs be identified on particular pages or groups of pages? How many groups are there? | | | | *See components listed previously.* | | | | *See components listed previously* | | | |
| Do any of the pages have response options to collect information in addition to baseline/follow-up measures? What data is collected? | | | | No. | | | | *Information on symptoms is collected in Doctors Questions.* | | | |
| 2. Accrued data. Data collected during an intervention. | | | | | | | | |  |  |  |
| 2.1. Self-report. Users’ self-reported responses collected across various stages of the trial. | | | | | | | | |  |  |  |
| When are self-report questionnaires collected? | Self-report was collected only at registration; no ongoing or follow-up questionnaires were administered | | | | *Baseline, every 4 weeks up to 24 weeks, during illness and 48 hour follow up.* | | | |  |  |  |
| What demographic information is available (e.g. age, gender, education)? | Age range, gender, role (e.g. school leader, teaching staff), school type, school location, school physical activity policy, source of information about the program | | | | *Baseline: Age, gender, qualification, household, smoker, alcohol, ethnicity.* | | | |  |  |  |
| Which measures are specifically related to the target behaviour and how often are they collected? | None | | | | *During illness, every 4 weeks & follow-up (see also external data): occurrence of illness and contact with all NHS.* | | | |  |  |  |
|  | | | | | | | | | | |  |
| Which measures of target behavioral determinants are collected and when? | | | None | | | | *Baseline & follow-up: health locus of control* [5]*, Krantz health opinion survey* [6]*, TPB* [1]*. When ill & 48hr: IPQ-R* [7]*, TPB* [1]*.* | | | |  |
| Are measures of health collected (e.g. conditions which may impact on target behaviour or are co-morbid) and psychosocial factors (e.g. anxiety, illness perception, motivation)? | | | None. | | | | *Baseline, when ill & 48 hour, & follow-up: (Physical) Mobility, self-care, usual activities, pain, anxiety/depression. Baseline (see also external data): health anxiety inventory* [8]*.* | | | |  |
| Are additional measures collected at follow-up (e.g. satisfaction, adherence)? | | | Nones | | | | *Follow-up: patient enablement* [10]*, website satisfaction, problematic experiences of therapy scale* [11]*.* | | | |  |
| 2.2. Log-data. Information automatically collected through engagement with an intervention. | | | | | | | | | | |  |
| What data is the software platform able to record? | | | Number of users, Number of new users, Number of sessions, Average sessions per user, Pageviews, Average pageviews per session, Average session duration, Average bounce rate, user navigation, time-based data, device information | | | | *Time and date, pages viewed & order, time spent on pages, self-report measures.* | | | |  |
| Are number, date and time of logins available by individual user? | | | *Yes.* | | | | *Yes.* | | | |  |
| Are individuals’ total durations of usage accessible? | | | Yes | | | | *Needs to be extracted.* | | | |  |
| Are the number and time of usage prompts recorded? | | | N/a | | | | *Overall scheduled timings for emails are available, but not sent times by individual.* | | | |  |
| Are there details for which pages were viewed, the sequential order and time spent viewing? | | | *Yes.* | | | | *Yes.* | | | |  |
| 2.3. External data. Data collected independently but alongside intervention usage. | | | | | | | | | | |  |
| How and where is the data collected? | | | Teacher surveys collected via Qualtrics surveys (baseline and endline) and short text messages several times each term. | | | | *Hand collected by the research team from users’ GP notes.* | | | |  |
| What data is collected? | | | *See methods* | | | | *Number of GP visits for RTI during trial, for year prior to trial, and co-morbid illnesses. Antibiotic prescriptions for RTI.* | | | |  |
| Which of these measures relate to or may impact on the target behaviour? | | | All of them may relate to the implementation level. | | | | *Number of GP visits for RTI during trial, for year prior to trial, and co-morbid illnesses.* | | | |  |
| 3. Contextual data. Data indirectly related to the running of the intervention which may be influential over usage and analysis. | | | | | | | | | | |  |
| 3.1. External factors. Structures and events which may influence participation in the intervention. | | | | | | | | | | |  |
| How are users recruited to the intervention? | | | Users were encouraged to register and access program resources on the website through dissemination efforts (e.g. social media, newsletters) with the help of *TransformUs* Partners (e.g. state department of education) | | | | *Recruited via GP.* | | | |  |
| Did any specific large-scale events, with the potential to impact on the intervention, occur during the period of the intervention? | | | N/A | | | | *N/a* | | | |  |
|  | | |  | | | |  | | | |  |
|  | | | | | | | | | |  |  |
| 3.2. Previous theory and findings. Results of behavioral analyses carried out during intervention development (e.g. logic models), and analyses of clinical outcomes if available. | | | | | | | | | |  |  |
| What are the hypothesized mechanisms of the intervention (e.g. as specified in the intervention’s logic model)? | | Providing teachers with free access to curriculum-aligned online resources is expected to increase their capability and confidence to deliver physical activity and sedentary behaviour programs, supporting adolescents to meet daily movement guidelines. | | | | *Users with low understanding of their symptoms will be less likely to consult their GP having viewed Doctors Questions and Common Questions. Users with low self-efficacy in their ability to self-manage their illness will be less likely to consult their GP having viewed Treatment Options.*  *Level of anxiety may be associated with usage.* | | | |  |  |
| Which factors are identified as important in qualitative research, and can they be related to the variables collected in the trial? | | N/A | | | | *N/a* | | | |  |  |
| Which variables are identified as relating to outcomes (e.g. behavioral determinants, theoretical constructs, health factors)? | | N/A | | | | *Analysis of the data from the RCT focused on the outcomes of GP contacts and antibiotic use* [13]*. No analyses were carried out for usage, behavioral determinants or personal characteristics.* | | | |  |  |
|  | |  | | | |  | | | |  |  |

*Stage 2*

| Selecting usage variables and generating research questions | | | | |
| --- | --- | --- | --- | --- |
| Generic questions | Intervention: *Physical Activity 4 Everyone (PA4E1)* | | Intervention: *Internet Dr* | |
| 1. Descriptions of usage variables. Which usage variables are relevant to the intervention and in which format (e.g. number of users/sessions, duration, percentage of total, dichotomous)? | | | | |
| Completing intervention/trial period (stage 1; 1.1 & 2.2). | 1) Number of registrations 2) Percent users access page beyond registration (general information, professional learning, resources) 3) Sessions 4) Page views 5) Time spent on pages | | *Number of users completing follow-up measures at 24 weeks. Number of users ill during trial period (logged in during illness, self-report at 4 week measures, from GP notes). Total number of pages viewed across the trial and pages with the greatest number of views.*  *Total time spent on pages.* | |
| Logins or sessions where the intervention was accessed (stage 1; 1.1 & 2.2). | N/A | | *Purpose of login: to complete baseline, 4 weekly measures or manage illness. Content viewed, number of pages and time spent when using intervention during illness.* | |
| Date of login and usage. | Session dates for each user groups. | | *n/a* | |
| Time of day of login and usage. | N/A | | *Proportion of usage outside vs during GP hours.* | |
| Days/weeks of usage (stage 1; 1.1 & 2.2). | Number of sessions website was accessed. | | *Number of times intervention content was accessed.* | |
| Response to prompts/notifications (stage 1; 1.1 & 2.2). | N/A | | *Number of users who logged in to illness follow-up at 48 hour.* | |
| Features/menu components used (stage 1; 1.1, 1.2 & 2.2). | 1) Number of registrations 2) Percent users access page beyond registration (general information, professional learning, resources) 3) Sessions 4) Page views 5) Time spent on pages | | *Number of users who viewed each of the linked menu components. Component most frequently viewed first. Doctors Questions: Dropout by page. Number of users moving back and forwards through pages. Split of users by symptom. Number of users who saw the advice pages, and type of advice given. Treatment Options & Common Questions: Number of pages viewed. Time spent in this component. Number of users who viewed each of the treatment options. Split of users by symptom.*  *Number of users viewing each video. Time spend on the video.* | |
| Revisiting components/features (stage 1; 1.1, 1.2 & 2.2). | *N/A* | | *Number of users re-visiting each component and frequency of revisits. Number of re-visits during illness. Number of users revisiting components with the same/different symptoms. Number of users who login when ill more than once.* | |
| Type of content/BCTs used (excluding administration pages) (stage 1; 1.2 & 2.2). | *See features/menu components* | | *See features/menu components* | |
| Completing ongoing measures (stage 1; 2.1 & 2.2). | *N/A* | | *Number of users completing monthly measures.* | |
| External device usage (stage 1; 2.3). | *Access via mobile vs laptop* | | *N/a* | |
| 2. Relationships between usage and participant characteristics. Are user’s demographic, physical or psychosocial characteristics at baseline related to intervention usage? | | | | |
| Are any characteristics at baseline related to usage? | Do teacher characteristics, school physical activity policies, and sources of information about the program influence engagement metrics (sessions, pageviews, time spent, and pages visited)? | | *Is having a co-morbid illness related to usage? Is age associated with different patterns of usage? Is level of anxiety at baseline associated with different patterns of usage?* | |
| Are any contextual factors related to usage (stage 1; 3)? | Dissemination strategies (active vs. passive) may influence website engagement. | | N/a | |
| Do high/low users differ by other usage factors? | 1) Does more total time spent lead to more sessions, pageviews, and greater likelihood of accessing training and resource pages? 2) Do higher pageviews indicate a greater tendency to access training and resource pages? | | *Is logging in when ill related to content previously viewed? Do users who self-report GP visits use the intervention differently to users who visited their GP but didn’t report it?* | |
| 3. Relationships between usage, behavioral determinants, and target behaviors. Which usage variables are associated with follow-up measures for target behaviour and behavioral determinants? Which usage variables help explain changes in behaviour across the intervention? | | | | |
| Are baseline measures for behavioral determinants/target behavior related to usage? E.g. Is the number of days the intervention is used for related to a behavioral determinant? Do users with low target behavior spend less time on the intervention? | N/A | | *Is the number of GP visits during year prior to trial related to usage? Are measures for health locus of control [*2]*, Krantz health opinion survey* [3]*, or TPB* [4] *attitudes/norms/intentions at baseline related to usage? Are measures for the IPQ-R* [5]*, TPB* [4] *beliefs/intentions at first login during illness related to usage during the period of illness (e.g. viewing content, completing 48hr follow-up).* | |
| Which usage variables are related to behavioral determinants/target behaviors and at follow-up? E.g. Do users who view a group of pages | Do those who access professional learning and resources tend to adopt and implement the program? | | *Is usage related to GP appointments? Is logging in when ill related to GP appointments? Is usage associated with the amount of times a user is ill* | |
| containing a specific BCT score higher/lower for the associated behavioral determinant? Is completing/not completing a particular component associated with target behavior at follow-up? Is the time spent on a session related to target behavior? | |  | | *during the trial? Are measures for health locus of control* [2]*, Krantz health opinion survey* [3]*, or TPB* [4] *attitudes/norms/intentions at follow-up related to usage? Are measures for the IPQ-R* [5]*, TPB* [4] *beliefs/intentions at 48hr related to viewing content during illness?* |
| Is usage associated with measures for acceptability/satisfaction at follow-up? E.g. Are high levels of satisfaction associated with accessing more pages? Do users with low satisfaction spend less time using external devices? | N/A | | *Are scores for patient enablement* [7]*, website satisfaction, or problematic experiences of therapy scale* [8] *related to usage?* | |
| Do users who report positive changes in behavioral determinants/target behavior from baseline to follow-up use the intervention differently to those who do not? | N/A | | *Did users with positive changes for measures for health locus of control* [2]*, Krantz health opinion survey* [3]*, or TPB* [4] *attitudes/norms/intentions use the intervention differently? Did users who reported increased positive beliefs at 48hr illness follow-up view different content during illness?* | |
| Are relationships between usage and outcome measures moderated by demographic, psychosocial or health factors? | Does the relationship between accessing professional learning and resources on the website and teacher implementation differ by school type or state? | | *Does anxiety moderate relationships between usage and GP visits? Does age moderate the relationship between usage and GP visits?* | |
| What level of usage is necessary for ‘effective engagement’? | How many pages need to be opened, how much time needs to be spent, and which pages should be accessed for adoption and implementation? | | *Did users need to see a specific amount or component of content, at a certain time in order to change their behaviour?* | |

*Stage 3*

| Preparation for analysis | | | | |  |
| --- | --- | --- | --- | --- | --- |
| Generic questions | Intervention: *Physical Activity 4 Everyone (PA4E1)* | | Intervention: *Internet Dr* | |  |
| 1. Resources | | | | |  |
| What is the timeframe for completing the analyses? | September – October 2025 | | *Oct 2018.* | |  |
| What resources are needed? E.g. additional research time, expertise | Website developer | | *Support with analyses, confounding variables (e.g. co morbid illnesses), and moderators. Support with visually exploring and extracting data.* | |  |
| Is a plan of analysis already available? How does the analysis plan developed using the framework compare to that plan? Are changes or updates needed? | No. | | *Primary analyses have already been carried out (see stage 1, 3.2), no further plan is available.* | |  |
| Is ethical clearance in place to carry out usage analyses? | Yes. | | *Yes.* | |  |
| 2. Selecting types of analysis and analytical software | | | | |  |
| Will the usage data be triangulated with qualitative data? | No. | | *No.* | |  |
| What analytical tools are available? | STATA SE 18.0 and Google Analytic | | *SPSS and LifeGuide Visualisation Tool (LVT).* | |  |
| Is there sufficient statistical power to answer the planned research questions? | *Metrics with larger responses (n=116) are likely sufficient to explore associations between teacher characteristics and website engagement, but caution is needed for metrics with fewer respondents.* | | *Analyses using whole intervention group are sufficiently powered, sub-group analyses will not be.* | |  |
| Can the selected measures of usage be analyzed using the available tools? Is bespoke software necessary (e.g. visualisation techniques)? | Yes. | | *Sequence of use of components and movement through Doctors Questions will need to be analyzed using LVT.* | |  |
| 3. Data preparation | | | | |  |
| When is the data available? | Now. | | *Now.* | |  |
| Is the data raw or has it been used/cleaned previously? | Raw log-data. | | *Raw log-data.* | |  |
| How many datasheets are there? Will these need to be amalgamated? | All relevant data are available from Google Analytics and can be downloaded according to the research questions. | | *5 datasheets: 4 log-data sheets, GP notes. LVT: Session details must contain important self-report measures from user data. Copy primary outcome measures, previous behaviour and co morbid* | |  |
|  |  | | *illnesses from GP notes. SPSS: all data for analysis must be available on one data sheet. Relevant measures from GP notes will be copied to session details, along with usage variables from user data and page durations.* | | |
| Is the data structured to work with the tools available? What formats are the datasheets in (e.g. excel, .csv) and will they need converting for analysis? | | Yes, data can be made available as descriptive statistics. | | *Datasheets are compatible with SPSS and LVT. Log data (session details, page flow, page durations, user data) is excel worksheet. These need to be converting to .csv for LVT. GP notes are SPSS.* | |
| What preparation does the data need (e.g. cleaning, anonymizing)? | | The descriptive statistics need running. | | *Data needs cleaning.* | |
| Are all variables readily available or will they need extracting/transforming/recoding? | | Some tidying and cleaning will need to occur, but most are ready. | | *User data datasheet has a row of data per login so that a user who has logged in 7 times will have 7 rows of data. However, session details datasheet has only 1 row per user. Data must be transformed into 1 row in order to copy across. Totals for time spent and pages viewed will need extracting.* | |
| Is the data in the right format to answer the research questions? Will it need adapting (e.g. continuous variables changed to categorical)? | | Most data are already in continuous variables, while some (e.g., pages visited) were converted into categorical variables to indicate access to professional learning and resources (yes or no). | | *Measures of behavioral determinants are measures as continuous variables. For comparisons of usage by high/low groups these* | |
